# Food Origin Authenticity Using Deep Learning and Citizen Science: Bananas Case Study

**DOI:** 10.3390/foods15101628

**Published:** 2026-05-07

**Authors:** Nikolaos Fragkos, Yamine Bouzembrak, Sara Wilhelmina Erasmus, Filipi Miranda Soares

**Affiliations:** 1 Information Technology, Wageningen University, Wageningen University and Research, 6700 HB Wageningen, The Netherlands; 2 Food Quality and Design, Wageningen University and Research, P.O. Box 17, 6700 AA Wageningen, The Netherlands; 3 Mathématiques, Informatique et STatistique pour l’Environnement et l’Agronomie (MISTEA), University of Montpellier, INRAE & Institut Agro, 2 Place Pierre Viala, Montpellier Cedex 2, 34060 Montpellier, France

**Keywords:** artificial intelligence, convolutional neural network, crowdsourced data, food fraud, RGB image

## Abstract

This study introduces an Artificial Intelligence (AI)-based proof-of-concept approach to tackle food fraud by using convolutional neural networks (CNNs) and citizen science-generated imagery to predict the country of origin of Cavendish banana cultivars (*Musa* spp.). A total of 6000 images were collected from iNaturalist, and a CNN classifier was trained to distinguish bananas sourced from six countries. Transfer learning was leveraged, and among nine pre-trained models tested, MobileNetV1 demonstrated the best trade-off between performance and computational efficiency. Following model fine-tuning, data augmentation was implemented to mitigate class imbalance and ensure a dense feature space. The model achieved an average accuracy of 0.86 with Monte Carlo Cross Validation and 0.77 with a 5-Fold Cross Validation. The final selected model attained a validation accuracy of 0.79. Accordingly, this study should be viewed as a foundational proof-of-concept demonstrating the potential of AI for origin detection at the cultivation stage. While the current evaluation framework reflects an early-stage experimental setting, the findings introduce a promising new dimension for proactive food fraud detection. Moving forward, this pipeline provides a foundation that can be expanded and independently validated.

## 1. Introduction

This study focuses on the application of Artificial Intelligence (AI), leveraging computer vision to tackle the issue of food fraud regarding origin mislabeling. The integration of AI into food science is increasingly recognized as a transformative tool for evaluating food structure, function, and authenticity [1]. Building on this momentum, the present study combines three key dimensions: food fraud, AI, and citizen science. Regarding the first dimension, food fraud is a multifaceted problem causing economic and health problems for society. Every food product needs to be correctly labeled as stated in the European Union (EU) Regulation No 1169/2011 where the general rule is applied, in which the indication of the country of origin or place of provenance is mandatory. Failure to indicate this might mislead the consumer as to the true country of origin or place of provenance of the food product [2]. The origin is a factor that can impact the price of the product and defines the profit margin of the fraudster as the fraud is mainly performed due to economic gain.

Several studies that focused on determining the country of origin of food products used laboratory-based data coupled with machine learning algorithms. Hou et al. (2024) [3] used ^1^H Nuclear Magnetic Resonance (NMR) spectroscopy with machine learning for origin determination of Longjing tea. Lia et al. (2021) [4] applied Fourier Transform Mid-Infrared Attenuated Total Reflectance spectroscopy for the authentication of Maltese virgin oil. Pérez-Rodríguez et al. (2019) [5] used spark discharge-laser-induced breakdown spectroscopy to determine brown rice origin authenticity. Across these studies, the implemented approaches made use of Support Vector Machine (SVM), Random Forest (RF) and Discriminant Analysis (DA) along with laboratory-generated datasets.

Our research focuses on bananas as the primary food product of interest, specifically investigating whether images of bananas associated with geolocated observations contain visual patterns that may support geographical discrimination. To achieve this, we leverage citizen science-generated data. We decided to focus on this specific fruit as it is considered the most ‘popular’ fruit, with its exports reaching 19.7 million tons and its imports reaching 19.0 million tons globally. The main exporting countries are Ecuador, Colombia, Costa Rica, and the Philippines in terms of annual performance [6]. The main importer of bananas is the European Union (EU-27), which imported 5.3 tons in 2024.

Concerning the price of the bananas, the average unit value of one ton of bananas originating from France in 2023 stood at 730 € while the price in Spain for the same quantity stood at 840 €. In Latin America the price ranged between 620 € and 690 € per ton. It is clear that the price is related to the country of origin of the fruit [7].

Our research focuses on the Cavendish cultivar (*Musa* spp., AAA group), the most widely traded commercial banana worldwide [6]. This cultivar dominates global markets, accounting for approximately 99% of international banana exports and 47% of total global banana production [8]. Globally, banana production reaches nearly 50 billion tons annually [9].

Citizen science constitutes the third dimension of this research. Citizen science according to the Oxford English Dictionary is the ‘Scientific work undertaken by members of the public often in collaboration with or under the direction of professional scientists and scientific institutions’ [10]. With citizen science, crowdsourced data can be gathered such as images to expand the scope and the granularity of data available to researchers. In this way, apart from contributing to science, citizens also become more aware of the topic they are engaged in.

In this study, geographical origin detection was performed by using Red, Green, Blue (RGB) images as inputs to a CNN. This approach aims to explore an innovative and alternative methodology by utilizing real-world data, specifically images captured by citizens, to identify the geographical origin of food products. To our knowledge, this method has not yet been applied. Based on the literature, geographical origin has so far been determined using techniques such SVM, DA, Neural Network (NN), regression, and RF with the aid of spectral, chemical, and genotype data. More recently, multimodal approaches have emerged where different types of data are combined; for instance, Gao et al. (2025) [11] combined RGB images with Near-Infrared (NIR) spectra to identify the geographical origin of apples. However, these methods typically rely on specialized spectral sensors and utilize images captured under controlled laboratory conditions with fixed lighting and standardized backgrounds.

Compared with laboratory-based approaches such as spectroscopy, chemical profiling, or multimodal sensor fusion, the present RGB-image approach offers potential advantages in accessibility, cost, and scalability because it does not require specialized instrumentation. However, laboratory-based methods benefit from controlled acquisition conditions and stronger analytical traceability. Accordingly, the present approach should be understood as a complementary, exploratory alternative rather than a direct replacement for established laboratory-based authentication methods.

In contrast to these laboratory-dependent approaches, the present study relies exclusively on citizen-captured RGB images and does not require controlled backgrounds or auxiliary sensor data. To our knowledge, this specific application of citizen science data for food origin authentication has not yet been explored. To this extent, the following research question was formulated:

Can the country of origin of bananas be accurately identified with AI by using RGB images captured by citizen scientists? 

By addressing this question, the study aims to fill an existing gap in the literature and explore the feasibility of using crowdsourced image data for determining the country of origin of a fruit as an alternative to conventional laboratory-based methods. Importantly, the aim of this study is not to provide a deployment-ready food authentication system, but to examine whether origin-related visual signals can be learned from citizen-generated RGB imagery, which can be further used for the authentication of country-of-origin. The study is therefore positioned as an exploratory proof-of-concept at the cultivation-observation level.

## 2. Materials and Methods

**Figure 1:**
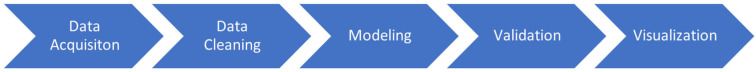
Schematic representation of the Data Science pipeline, adapted from Biswas et al., 2022 [12].

The structure of methodology used in this study is based on the concept of the Data Science pipeline [12] as illustrated in Figure 1.

**Figure 2:**
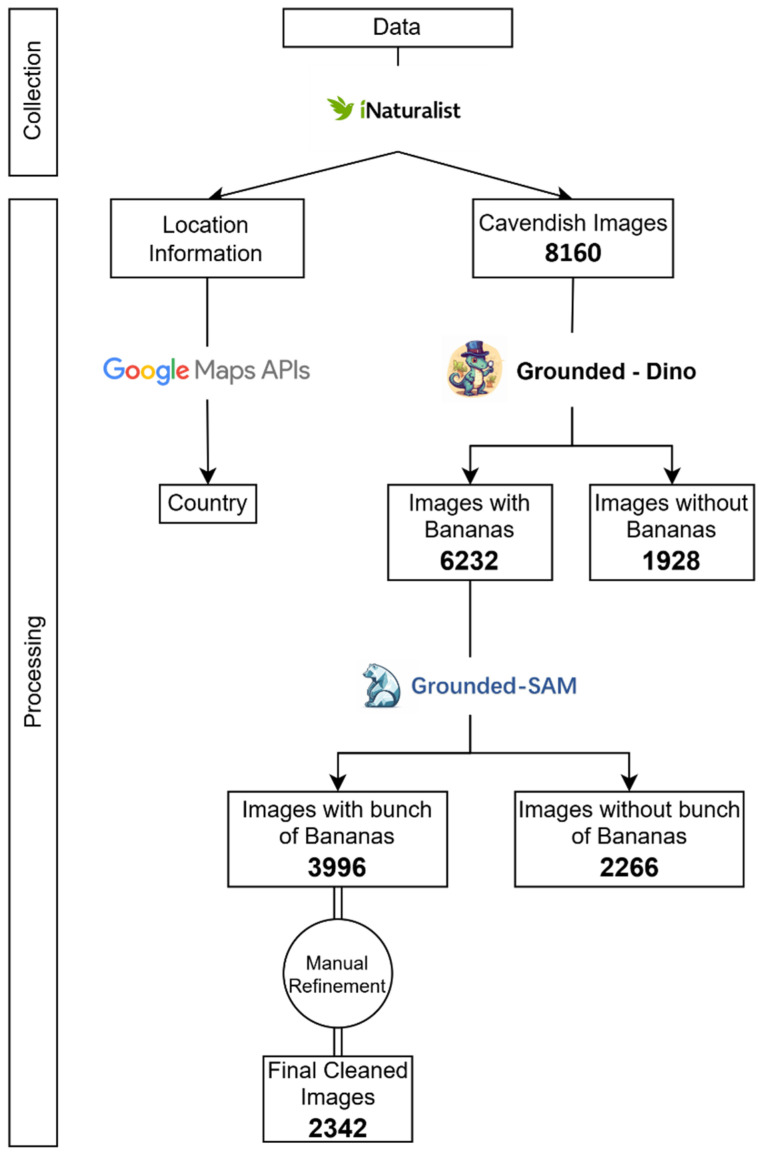
Comprehensive data processing workflow illustrating the parallel refinement of location and image data.

The overall methodology followed in this research is summarized in Figure 2 below:

### 2.1. Data Collection

The data used in this study were collected from iNaturalist (https://www.inaturalist.org/ (accessed on 15 November 2024)), an online network of people sharing biodiversity information to support learning and research on biodiversity. iNaturalist is a nonprofit organization based in the United States and enables users to explore a wide range of taxa, including both wild species and cultivated plant varieties. Users can contribute to the repository by uploading images, referred to as “observations,” which may be accompanied by metadata such as species identification and geographic location [13].

Although iNaturalist primarily focuses on documenting observations of wild, non-cultivated organisms, the platform has also been used by initiatives targeting cultivated plants. For example, the Urban Orchard project (Pomar Urbano, in Portuguese) in Brazil leverages iNaturalist to collect observations of more than 400 cultivated fruit species, including both native and introduced species (including *Musa* spp.) [14,15].

In this case study, we focus on the Cavendish cultivar of the banana plant (*Musa acuminata*). To extract the images, we utilized the website’s Application Programming Interface (API), which provided a structured and efficient means of accessing the data. The API was accessed with Jupyter Notebook v7.5, employing Python version 3.12.7 as the programming language. At that time, a total of 8252 observations were collected for the selected cultivar. All these observations were accompanied by a location label. The output of the extraction procedure was a Comma-Separated Values (CSV) file including the Uniform Resource Locator (URL) of each and the location. The data were collected on 15 November 2024. The API’s (https://api.inaturalist.org/v1/observations (accessed on 15 November 2024)) endpoint was used to access the observation data stored in the database of iNaturalist. The parameters for the query are configured as follows:

1. taxon_id: This parameter is set to ‘140907’, which is the unique identifier of the Cavendish cultivar in the iNaturalist database. This ensures that only relevant observations to this specific taxon are captured.
2. page: The value is set to 1, indicating that the query will start with the first page of the results.
3. per_page: This parameter is assigned to the value of 200, which is the maximum value of the results that can be returned to a single page.
4. geo: This parameter is set to ‘true’, and it was applied to enforce the presence of geolocation metadata.
5. quality_grade: This parameter is set to ‘Research Grade’. This status confirms that the observation has been vetted by at least two independent community experts who have reached a consensus on the species identification.

The selection of the ‘Research Grade’ filter was critical for ensuring label validity, as it requires expert consensus to confirm the species identification. Beyond botanical accuracy, this filter ensures that the images provide sufficient visual context, showing the fruit attached to the plant in a natural setting. While seeing the plant in its growing environment strengthens the assumption that the GPS coordinates relate to a cultivation site rather than a distribution point, it does not fully validate it. Therefore, the geographic labels in this study should be interpreted as geolocated botanical observations rather than definitive proof of commercial product origin.

After obtaining our first dataset, a check for missing values is conducted, reducing the number of data points from 8252 to 8160. Even though we selected images with geographical data during the data collection through the API, these data were not extracted correctly, resulting in missing values. Some observations either had a location that was empty, or the URL of the image could not be retrieved successfully.

The final step of processing the dataset involves the linking of the downloaded images with their label regarding the geographical location. The unique identifier of each image was parsed from its source URL using Regular Expressions. The unique numeric identifier of each image was parsed from its source URL and used to establish an absolute local file path.

### 2.2. Data Processing

**Figure 3:**
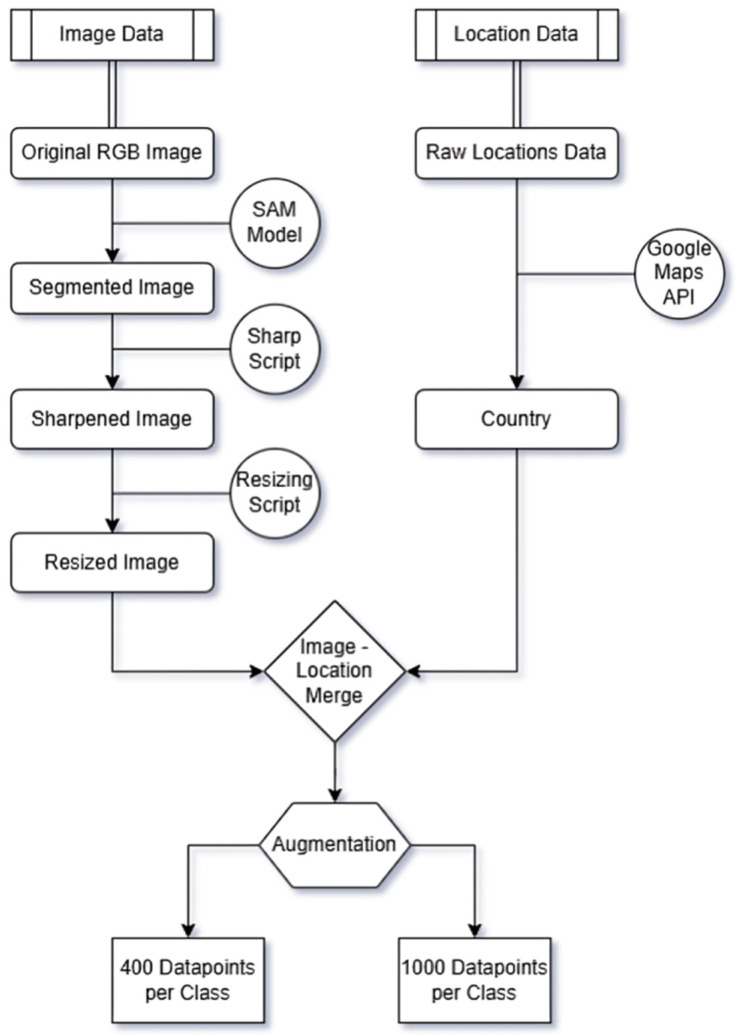
Detailed image preprocessing and augmentation pipeline.

After gathering the initial, unprocessed version of our data, the subsequent step involved data processing. The data processing section is a crucial step in our research, encompassing two distinct branches: the processing of image data and the processing of location data, as shown in Figure 3. It should be noted that the geocoding of the initial dataset (8160 locations) and the manual refinement of the image set were conducted as parallel processes. This approach was adopted to optimize research time, allowing for the independent processing of complex location strings while simultaneously performing manual visual quality checks on the raw images. The final dataset represents the intersection of these two workflows, where only high-quality images with successfully resolved geographic coordinates were retained.

#### 2.2.1. Image Processing

**Figure A1:**
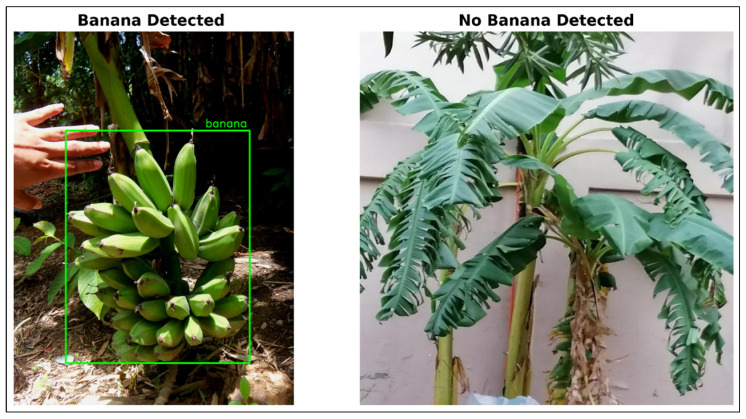
Automated object detection using the Grounded-DINO model.

The dataset consists of real-world images captured in natural environments under uncontrolled conditions. It not only includes images containing bananas in bunches or as single units, but also some images of banana trees without any visible bananas. To account for this variability in images, they must be classified into two categories: those containing bananas and those without them. For this separation, we utilized the Grounded-DINO model [16], a state-of-the-art object detection model that can detect any object based on textual prompts. The detection process is shown in Figure A1 in Appendix A.

To separate images containing bananas from those that do not we used the term ‘banana’ and we developed a script that loops over all the images in a folder and then classifies them into two separate folders based on our criteria for containing bananas or not.

This model utilizes the PyTorch v2.5.0 library; it can run either on the Central Processing Unit (CPU) or the Graphics Processing Unit (GPU) of the system. For our setup, the GPU was employed to accelerate image processing. The image-classification procedure was performed in multiple stages. A custom script was developed to iterate over each image and check whether it contains the term ‘banana’. If the term is detected, the image is copied to a folder designated for images containing bananas: otherwise, it is stored in another folder for images without bananas.

Once the images were divided into their respective folders, a manual verification was performed to assess the accuracy of the classification. The incorrectly classified images from the ‘without bananas’ folder were copied into the ‘with bananas’ folder to ensure accurate categorization.

Overall, the model achieved an accuracy of approximately 0.71, (Appendix A Table A1). Notably, the model produced a high number of false positives, frequently confusing images of banana plants (without actual bananas) with those containing fruit. To prevent this noise from entering the classification pipeline, the manual verification step was critical.

Then, the segmentation process was initiated. The segmentation was performed with the Grounded-Segment Anything Model (SAM) model, an extension of the Grounded-Dino model used before. Grounded-SAM utilizes a textual prompt to detect an object and segment it from the rest of the image. For the segmentation, the model searched each image for the term ‘bunch’ instead of ‘bunch of bananas’, as each one of the images already contained bananas. Furthermore, the decision to use this term arose from an observed issue where the term “bunch of bananas” produced multiple bounding boxes instead of one. By using the simplified term “bunch”, the model consistently generated a single bounding box around the detected object with high accuracy.

**Figure A2:**
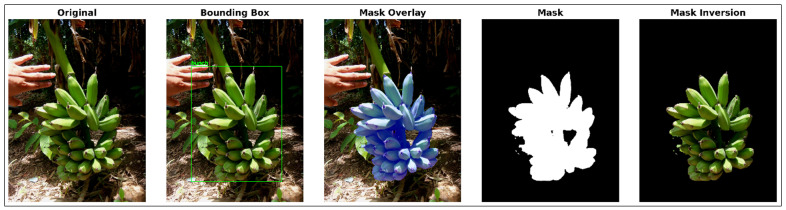
Sequential segmentation process utilizing the Grounded-SAM.

The segmentation loop iterated over all images, detecting the desired object, generating a mask for the object, and then applying mask inversion. In this way, the final output was an image where only the segmented object was visible, with the background being black as shown in Figure A2 of Appendix A.

To quantify the precision of the segmentation pipeline and ensure robustness against occlusion (e.g., leaves, branches), a post hoc visual audit was conducted on a random subset of 100 processed images. The audit aimed to verify that the ‘Mask Inversion’ step consistently removed background noise while retaining the relevant fruit morphology.

The analysis revealed a segmentation success rate of 98%. In the vast majority of cases, the banana bunch was correctly isolated, and all background elements were completely eliminated. While Figure A2 illustrates an intermediate step where the pseudo-stem is visible, the final masked output consistently excludes non-botanical artifacts. This high success rate confirms that the pipeline effectively filters out environmental noise.

#### 2.2.2. Image Uniformization

After having processed the images, it was observed that some images were of low quality. To enhance their clarity and usability, a preprocessing and refinement script was developed. The procedure was implemented using Python with the OpenCV library. Two directories were defined: the input directory, which contained the raw images, and the output directory, which stored the processed images. The pipeline is as follows:

1. Grayscale Conversion: Each image is converted to grayscale to make the application of thresholding and morphological operations easier.
2. Thresholding: A binary mask is created to isolate the regions of interest based on pixel density. Through this step, foreground objects are separated from the background.
3. Morphological Operation: For the cleaning of the mask and the removal of noise, morphological operations are applied using an elliptical kernel. This process refines the mask by closing small gaps and removing extraneous elements.
4. Contour Detection and Filtering: Contours in the cleaned mask are detected, and only large contours, indicating significant foreground regions, are retained. This step ensures that small, irrelevant regions are excluded.
5. Foreground Extraction: Using the refine mask, the foreground image is isolated. This foreground is then combined with a black background to produce the final processed sharpened image.

**Figure A3:**
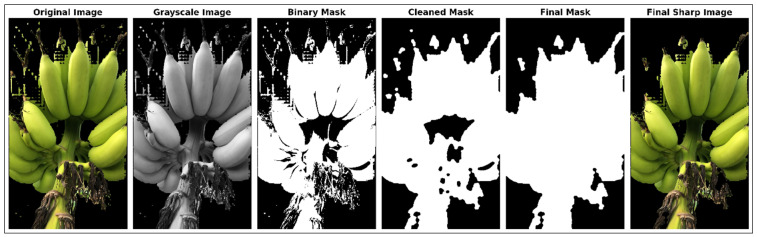
Image uniformization and sharpening workflow.

The objective of this script was to transform raw images with visual noise into a clean and refined version suitable for the modeling stage (Appendix A Figure A3).

After having sharpened the images, variations in size and zoom levels were still present. Some images appeared more zoomed in than others and there were some unnecessary black areas in the images. For this reason, a cropping script was developed to eliminate the black regions and standardize the images. As before, two directories were used, one containing the sharpened images and the other one cropped images. The cropping logic works as follows:

1. A completely black image of the same size is created as input for comparison.
2. The difference between the input image and the black one is calculated. This difference highlights all non-black areas in the image.
3. The bounding box of the non-black region is determined with the “getbbox” function. This box defines the smaller rectangle including all the non-black pixels.
4. The image is then cropped around the box with only non-black pixels.

**Figure A4:**
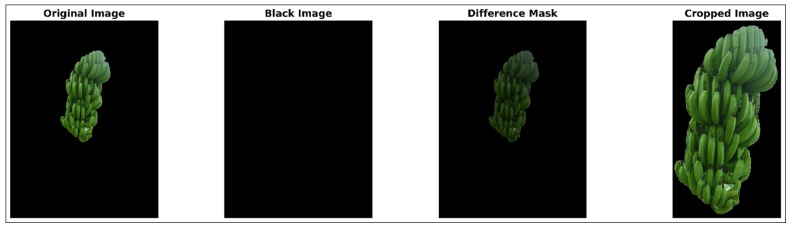
Automated cropping procedure designed to eliminate excess background space.

This step ensures that the images are optimized by removing extraneous black regions, improving the quality of the images (Appendix A Figure A4).

**Figure A5:**
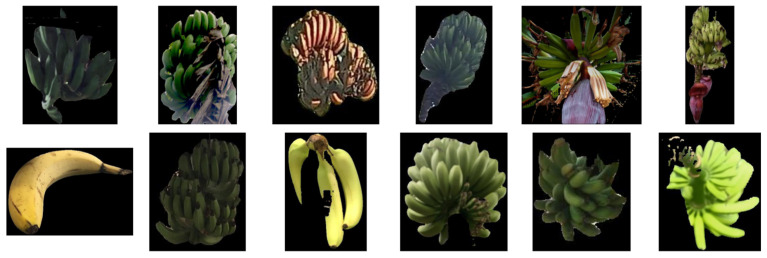
Representative examples of images excluded during the manual refinement stage.

To ensure the high quality of the image dataset, a manual filtering step was performed afterwards. During this step, images that were deemed unsuitable for further analysis were systematically removed. These images were excessively shaky, blurred, or of poor quality as they would negatively affect the accuracy of our model and would introduce noise (Appendix A Figure A5). The images that were included were clear, well-framed, and free of distortion. Also, there were images containing single units of bananas or they included a stem deriving from the bunch of bananas. Those images were also removed as they were adding complexity and only images with a bunch of bananas were kept. This step could not be automated as it would fail to detect poor framing or composition issues. Manual inspection ensures a higher accuracy in detecting problematic images that could be overlooked by an automated procedure.

Lastly, after finalizing the visual dataset, the images need to be evenly resized to ensure compatibility with the DL model. Since the model receives only images of fixed dimensions, a script was developed to resize each image to a target size of 224 × 224 pixels while maintaining its aspect ratio to prevent stretching the images vertically or horizontally. Each image was resized to fit within the target without distortion using the LANCZOS resampling filter which allows high-quality resizing using sinc-based interpolation to preserve image details and minimize artifacts during scaling. It is effective in downscaling images as it reduces aliasing and ensures smooth transition in detail.

After resizing, some images did not perfectly fill the desired frame. To maintain uniformity, black padding was added to fill any remaining space instead of zooming, which could result in lower-quality images. This padding was calculated dynamically based on the difference between the resized image dimensions and the target size. The padding was also added symmetrically to ensure the object remained centered.

#### 2.2.3. Location Processing

The second part of the dataset represented the geographical location of each observation. These data were recorded in free-text format and exhibited significant variability, including inconsistent formatting, mixed languages, and encoding errors. Table 1 contrasts this raw input, presenting the original inconsistent characters with the final standardized output used for analysis.

Initially we attempted to process the data using regular expressions to extract the country and the state where each image was taken. However, identifying the state proved to be very challenging, as it was missing in more than half of the observations. Consequently, we decided to focus only on extracting the country. Given the fact that our initial dataset contained nearly 8000 images, manual refinement was not a feasible option. For this reason, we explored three different approaches to process the data effectively.

The first approach was the extraction of the location data from the Photon API (https://photon.komoot.io/ (accessed on 13 January 2025)). A script was developed so that the tool iterates over each location registry and returns the value of “country”. Due to the wide variability of the location data, also mentioned above, the tool did not work as expected as a lot of countries were wrongly extracted or they were not extracted at all.

The second approach involved the Nominatim API (https://nominatim.org/ (accessed on 13 January 2025)), a geocoding service based on OpenStreetMap data. There was a time limit according to the usage policy of the API. It was also taking a lot of time to process, and it was impossible to use parallel processing as it was contradicting the policy of the API. Lastly, a manual approach was initiated. An inverse dictionary was built to replace variations of country names and abbreviations with a standardized format (inverse_country_mapping = {‘Mexico’: [‘MX’,’México’,’Meksyk’], etc.). Then we extracted city names and country codes from the GeoNames dataset, building a dictionary linking country codes to associated city names, and saving this dictionary into a pkl file. There were a lot of unmatched locations due to different language usage, so the googletrans (https://pypi.org/project/googletrans/ (accessed on 13 January 2025)) library was used for language detection and then the language was mapped to the respective country. For example, the address ‘622 台灣嘉義縣大林鎮林子前路’ was detected as Chinese and consequently mapped to the country code ‘CN’. However, this linguistic approach lacked regional precision (e.g., distinguishing Taiwan from mainland China), which contributed to our decision to switch to the Google Maps Geocoding API. Finally, pycountry (https://geopy.readthedocs.io/en/stable/ (accessed on 13 January 2025)) was used to verify and standardize unresolved locations, filtering and displaying missing cases for refinement. However, despite these extensive efforts, a significant portion of the information remained incomplete. The high variability due to non-standardized formatting and the presence of multiple languages rendered the whole manual process obsolete.

To overcome the limitations of the manual approach, the Google Maps Geocoding API (https://developers.google.com/maps (accessed on 13 January 2025)) was utilized for the extraction of the country for each location in the dataset. Google Maps API is a service that can convert addresses or place IDs to latitude/longitude coordinates and vice versa. So, we developed a script that goes over every location data point and returns the name of the country. The name of the country in the API was named “country” and the state was named “administrative_area_level_1”. This approach ensures that multilingual regions are handled correctly based on geographic coordinates rather than linguistic heuristics. In cases where no valid values were returned or an error had occurred, the fallback value such as ‘Address not Found’ was used to ensure consistency. Again, multithreading was used to reduce overall runtime significantly. Given the budget constraint and to minimize redundant API calls, measures were taken. Periodic saving of the dataset was performed to avoid unnecessary queries for the same locations. Specifically, every ten processed addresses the dataset was saved to a file, ensuring that progress was continuously stored.

Finally, a manual review was performed on the processed location data to handle cases where the value “Address not Found” occurred. Specifically, there were 70 such cases. Through the manual check, 57 of the values were handled and were standardized, leaving the dataset with only 13 cells containing the “Address not Found” value. These values were not processed with the Google Maps API or verified with the manual check as they were considered ambiguous. This means that they referred to places that could exist in multiple countries due to the same name, a fact that made the determination of the exact location difficult. Another discrepancy in the location dataset was the presence of country abbreviations in some cells, which resulted in the value “Address not Found”. To address this issue, a check was implemented in the script to examine the last two characters of each entry. Based on this information, the country value was assigned accordingly.

#### 2.2.4. Merging Labels and Images

To ensure proper alignment between the images and their corresponding location data, a matching process was implemented. Each image is stored in the dataset with a unique identifier, derived from its URL and follows a standardized format “segmented_xxxxx.jpeg”. This naming allows a straightforward mapping between images and their respective geo-data entries.

Using this unique identifier, the script scans the entire folder containing the images and cross-references them with the dataset. If an image is present in the folder, it is matched with its respective label in the dataset. The dataset now includes two key columns: the full file path and the corresponding label. Any rows where the image file is missing from the folder are removed from the dataset to maintain data integrity and include only valid images. In this way, the dataset is almost ready for usage from the model as every local path to each image is accompanied by a geo-label.

#### 2.2.5. Final Dataset Creation

##### Class Balance Check

With the final dataset prepared, it is crucial to analyze the distribution of the data points in the classes, as well as the corresponding image counts for each class. The dataset comprises 120 distinct classes/countries; however, a significant portion of these classes have only a single image. Table 2 below illustrates the top 20 classes ranked in descending order based on the number of images they contain. This dataset in its current state exhibits a notable class imbalance. This imbalance could add bias to our model during the training phase, resulting in a model being overly trained in classes with a higher number of images and consequently a higher representation, while struggling to accurately predict or generalize for classes that are underrepresented. This issue will not only affect the model’s performance negatively, but it will also compromise its reliability.

##### Data Reduction

To address this issue of class imbalance, we will apply a data reduction strategy by removing certain data points. This approach involves creating three distinct subsets of the datasets based on the image count per class. The three subsets will be defined as follows:

1. *Subset 1*: Includes classes with an image count of 50 images and above.
2. *Subset 2*: Includes classes with an image count of 100 images and above.
3. *Subset 3*: Includes classes with an image count of 150 images and above.

Through this subset creation, the aim is to conduct a comparative analysis of the model’s performance under varying degrees of class balance. In Table A2 in Appendix A, the subsets are presented.

##### Data Augmentation

Following the formation of the filtered datasets, the reduction in data points posed a challenge for CNN training because the remaining classes were imbalanced and limited in size. As reported in the literature, deep learning architectures trained on limited datasets are highly susceptible to overfitting and instability [17]. In addition, a pre-augmentation split based only on the original observations would have yielded a limited number of test images per class, thereby constraining the statistical stability of class-wise error estimates [18,19]. However, enlarging the apparent test set through augmentation prior to splitting does not provide independent evaluation samples and therefore does not eliminate the risk of data leakage.

While algorithmic approaches such as class-weighted loss functions or focal loss can mitigate class imbalance during optimization, they do not increase the visual diversity of the observed samples. For this reason, data warping was selected on the basis of prior image-classification literature as a suitable augmentation strategy for enriching within-class variation under limited-sample conditions. Data warping introduces geometric modifications within the image space, and in this study random rotations, shear, flipping, and zoom were applied to simulate plausible natural variation in image appearance [20]. In the present study, the purpose of data warping was to expose the model to a broader range of geometric transformations rather than to create statistically independent observations. Accordingly, because the augmented samples still originate from the same source images, applying augmentation before dataset splitting retains the possibility of leakage across training and evaluation subsets. By applying augmentation before the dataset split, we explicitly acknowledge the potential risk of data leakage, as augmented variants of the same original image may appear in both the training and evaluation sets. Consequently, the performance metrics reported in this study should be interpreted as an assessment of the model’s ability to generalize within this high-variance, augmented domain rather than as a definitive measure of independent out-of-sample generalization. Following the evidence provided by Wong et al. 2016 [21], which demonstrates that geometric warping outperforms synthetic oversampling, we applied high-variance spatial distortions to increase within-class visual variability under limited-sample conditions.

The augmentation was executed via a custom Python script utilizing the Keras ImageDataGenerator (https://github.com/keras-team/keras/blob/v3.3.3/keras/src/legacy/preprocessing/image.py#L949-L1547(accessed on 21 January 2026 )) class. The script iteratively processed each class. In each iteration, an image was randomly selected and modified through the parameter suite detailed in Table 3, creating a new data point while preserving the original label. Two augmentation approaches were deployed:

1. Augmentation to 400 total data points per class.
2. Augmentation to 1000 total data points per class.

We deliberately chose not to alter the color properties, as the dataset already exhibited significant color variation due to the different angles of sunlight on the bunches. By introducing additional color variations, it could have resulted in unnecessary distortions. By preserving the color variations, we ensured that the augmented data remained representative of the real-world conditions. Building on the subsets defined in the Data Reduction section previously, this strategy ensured complete class balance for the final modeling phase. Specifically, we took the three filtered subsets—Subset 1 (1585 original images), Subset 2 (1264 original images), and Subset 3 (1033 original images)—and augmented each class to reach uniform targets of 400 and 1000 images. Following the augmentation process, the following datasets were created:

• augmented_50 × 400 and augmented_50 × 1000: Based on Subset 1, with 400 images and 1000 images, respectively.
• augmented_100 × 400 and augmented_100 × 1000: Based on Subset 2, with 400 images and 1000 images, respectively.
• augmented_150 × 400 and augmented_150 × 1000: Based on Subset 3, with 400 images and 1000 images, respectively.

#### 2.2.6. Modeling

This section outlines the development of the modeling framework designed to predict the geographical origin of a banana based on an RGB image. To streamline the model development part, we employed transfer learning, leveraging a pre-trained model that was subsequently fine-tuned to address the needs of our research. For the model development process, the Keras module from TensorFlow was utilized.

##### Model Selection Criteria

The selection of the model was based on two primary key factors: the computational resources available and the complexity of the model. To ensure computational feasibility, we focused on lightweight models, with a size below 50 MB and fewer than 10 million parameters. These criteria were set to achieve a balance between performance and computational efficiency. A set of pre-trained models meeting these criteria is summarized in Table A3 in Appendix A.

##### Benchmarking Pre-Trained Models

All the models were benchmarked across all subsets of the dataset in a preliminary screening phase. To maintain computational feasibility while testing multiple architectures, training was limited to 10 epochs. Given the use of Transfer Learning with pre-trained ImageNet weights, this duration was sufficient to differentiate between models capable of rapid adaptation and those failing to converge on the specific dataset. Models showing significant learning capability (high accuracy) were advanced to the final selection stage, while those failing to improve beyond random guessing were discarded. The results are summarized in the Table A4 in the Appendix A, where:

• “50 × 400”, “100 × 400”, “150 × 400” refer to the augmented version of the respective datasets, with each class containing 400 images.
• “50 × 1000”, “100 × 1000”, “150 × 1000” refer to the augmented version of the respective datasets.

The benchmarking results showcased significant variation in model performance across datasets. Notably, MobileNet, MobileNetV2 and Densenet121 demonstrated superior accuracy compared to other models. On the contrary, models such as EfficientNet and its variations achieved uniformly low performance. This may lead to the conclusion that models with simpler architecture and fewer parameters may generalize better with limited data for our case.

##### MobileNet Architecture Selection

Since the MobileNet models were closer in terms of accuracy, we benchmarked them against all the existing models of the MobileNet architecture to provide a thorough comparison and identify the most suitable model. The results of this comparison are presented in Table 4.

Based on the benchmarking results, MobileNetV1 emerged as the best-performing model, achieving the highest test accuracy of 0.74 with a relatively low test loss of 0.75.

##### MobileNetV1 Architecture

The MobileNet model belongs to a category of small, low-latency models for mobile and embedded vision applications. Its efficiency is primarily due to the use of depth-wise separable convolutions, a form of factorized convolutions which factorize a standard convolution into a depth-wise convolution and a 1 × 1 convolution called a pointwise convolution [22]. Instead of using regular convolution when a filter is applied to the entire depth of the image, each channel is processed independently. After the depth-wise convolution is done, then the pointwise convolution is applied across all channels to combine the information. In this way, the relationship between the channels is captured. Specifically, the MobileNet architecture is cited in detail in Supplementary Materials Table S1.

##### Customization of the Base Model

In our case, the base model was the pre-trained MobileNetV1, serving as the feature extractor, which was trained on the ImageNet dataset. To adapt the model for the specific task of predicting the geographical origin of bananas, several custom layers were added after the base model with the respective order as cited below:

1. Global Average Pooling Layer (GlobalAveragePooling2D (GAP)):

This layer reduces the spatial dimension of the feature maps generated by the base model by computing the average value of each feature map. This layer reduces the number of parameters, helping to prevent overfitting while maintaining the most important features. It converts the 2D feature maps into a single vector, making the data suitable for the subsequent dense layers.

2. Fully Connected Layer (Dense (128, activation = ‘relu’)):

A dense layer with 128 neurons is added so that the model can learn high-level representation from the pooled features. The ReLu activation function is used introducing non-linearity, allowing the model to learn complex patterns in the data.

3. Dropout Layer (Dropout (0.3)):

To reduce the risk of overfitting, a dropout layer with a rate of 0.3 is applied. This means that 30% of the neurons are randomly deactivated during each training iteration.

4. Output Layer:

The final layer is a dense output layer, where the number of neurons corresponds to the number of unique classes, in our case the distinct countries. The SoftMax activation function is used to convert the raw output scores into probability distribution across all classes. The class with the highest probability is selected as the model’s final prediction.

**Figure 4:**
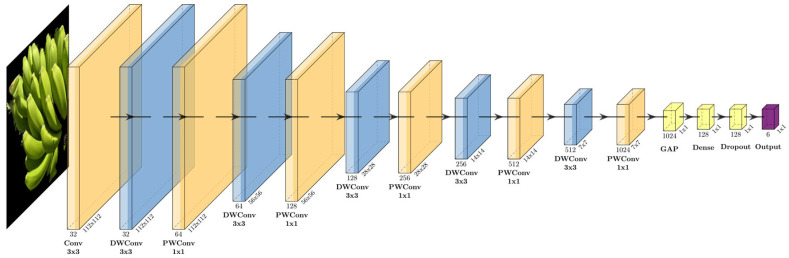
Schematic of the customized MobileNetV1 architecture utilized for classification.

In this study, the full MobileNetV1 architecture is implemented and used for our model. However, for the purpose of visualization and clarity in this document, a simplified version of the architecture is presented as shown in Figure 4. This simplified representation abstracts repeated depth-wise separable blocks and more detailed layer configurations; it highlights the overall flow from the initial image, through the base model, to the global average pooling and finally to the classification head.

##### Baseline Model Configuration

To establish a performance benchmark, a baseline architecture was implemented. The architectural parameters for this baseline are summarized in Table 5.

The input images were resized to 224 × 224 pixels with 3 color channels (RGB) as this is the image size that the MobileNet model can receive. The loss function that is used is suitable for multi-class classification tasks, where labels are in the form of integers. To prevent overfitting, early stopping was implemented with a patience of 3 epochs, meaning that the training will stop if there is no improvement for 3 consecutive epochs and the best model weights are restored. Lastly, the learning rate was reduced by a factor of 0.2 if no improvement in the validation performance was observed for 2 consecutive epochs. The rest of the parameters employed in this study were selected as a baseline configuration and represent commonly adopted choices for CNNs. After obtaining the basic configuration and the dataset consisting of 6 classes with a total of 6000 data points, the next stage is the fine-tuning process.

##### Fine-Tuning

The fine-tuning process involved optimizing key hyperparameters: batch size, dropout rate, learning rate, and dense units. A systematic grid search was developed to explore all possible combinations of the hyperparameters above. All four hyperparameters had three possible values each, resulting in 81 total possible combinations. The entire fine-tuning procedure took approximately two hours to complete.

To determine the optimal network depth, we conducted a functional ablation study by comparing classification heads with varying capacities (64, 128, and 256 units) while keeping other parameters fixed. As shown in Table 6, this analysis revealed that lower-capacity heads (64 units) resulted in significant underfitting with a validation accuracy of only 49.25%. Increasing the capacity to 128 units improved performance to 77.5%, but the model still lacked the representational power to fully capture morphological nuances. The configuration with 256 units achieved the highest performance (82.17%) with the lowest validation loss (0.498), representing the optimal trade-off between convergence and generalization.

The best-performing model configuration is summarized in Table 7. The comprehensive results of the hyperparameter grid search are provided in Supplementary Materials Table S2.

##### Epoch Adjustment

The determination of the optimal training duration was approached as a two-stage process. Initially, a fixed benchmark of 10 epochs was already used to evaluate the preliminary convergence of the multiple models. As shown in Table 8, while 10 epochs established a baseline for model comparison, the relatively high test loss (0.498) indicated that the model had not yet reached its global minimum. To allow for full convergence without the risk of overfitting, we implemented an automated training strategy with a 50-epoch limit governed by an early stopping mechanism. This mechanism utilized a patience window of 5 epochs, programmed to terminate execution if the validation loss failed to show significant improvement.

Under this protocol, training automatically terminated at epoch 18. This resulted in a significant reduction in test loss (from 0.498 to 0.095) and improvement in accuracy (from 0.8217 to 0.8667). 

##### Evaluation Metrics

In this section, the evaluation metrics used to evaluate the performance of the model are described in detail. The evaluation metrics that are used is a confusion matrix, which is presented as a heatmap, where the color intensity indicates the magnitude of correct and incorrect classifications. Brighter colors denote higher values, making it easier to detect patterns of misclassification. Other metrics are overall accuracy alongside per-class precision, recall (Sensitivity), the F1-Score, and the Multi-Class Receiver Operating Characteristic (ROC) Curve. These metrics are critical for assessing performance on the high-variance, citizen science dataset where simple accuracy may hide class-specific weaknesses. To ensure a robust performance estimation, two Cross-Validation strategies will be implemented. A 5-fold Cross-Validation, where the dataset is partitioned into five distinct folds; the model is trained five separate times, with each fold serving as the test set once. Complementary to this, Monte Carlo Cross-Validation (MCCV) is utilized to perform multiple iterations of random train–test splits, allowing for a robust assessment of the model’s performance stability and variance across different random samplings [23]. To move beyond numerical accuracy and provide a qualitative understanding of the model’s logic, we employ Gradient-weighted Class Activation Mapping (Grad-CAM) [24]. Grad-CAM is a visualization technique that highlights regions of an input image that are most influential in the decision-making process. It computes the gradient of the target class score with respect to the final convolutional layer. Then, it weighs the importance of each feature map and then generates a heatmap over the original image to highlight critical regions. In this study, Grad-CAM heatmaps were generated for each class by aggregating data from individual observations. By summing the pixel weights to produce a composite visualization, we were able to identify the systematic morphological features that the model prioritized when distinguishing between different geographical origins.

## 3. Results

**Figure 5:**
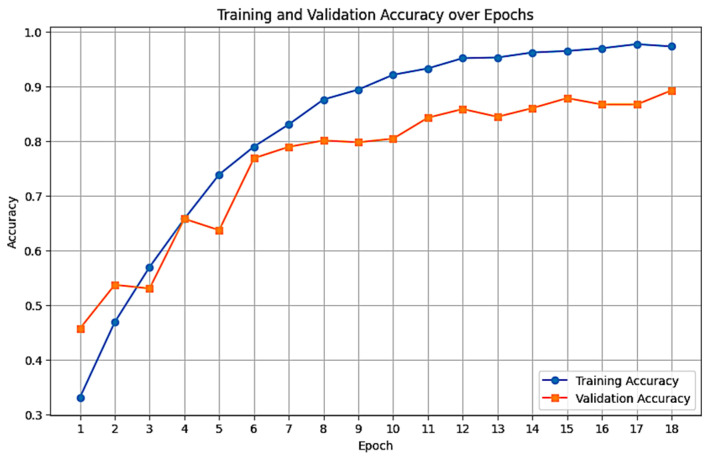
Training and validation accuracy progression over 18 epochs.

**Figure 6:**
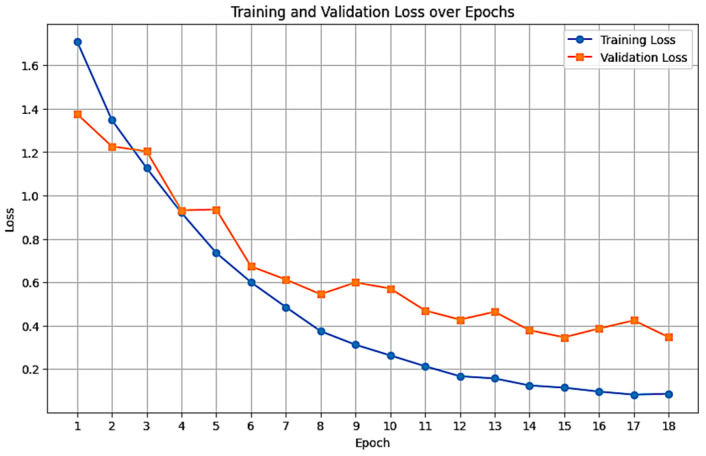
Training and validation loss trajectories during the training phase.

Under the present experimental setup, the model was able to discriminate images of banana bunches across six countries with moderate-to-high apparent accuracy. However, because augmentation was performed prior to dataset splitting, these values should be interpreted as upper-bound performance estimates under non-independent evaluation conditions rather than as true measures of out-of-sample generalization. As the data points are shuffled every time that the model is trained, to ensure that the model is robust and can generalize well, the model was trained and evaluated across five independent runs (MCCV), yielding and average accuracy of 0.86 with a standard deviation of ±0.012. The final optimized hyperparameters are presented in Table 9:

Figure 5 illustrates the training and validation accuracy across 18 epochs. Both training and validation accuracy are increasing with a steady rate, reaching 0.97 and 0.89, respectively, by the final epoch. Also, Figure 6 presents the training and validation loss, demonstrating a decreasing trend which stabilizes around the 15th epoch. An early stopping mechanism (patience of 5 epochs) was implemented. While training continued to epoch 23 to confirm convergence. Validation accuracy ceased improving after epoch 15; thus, optimal weights from epoch 15 were restored to prevent overfitting.

**Figure A6:**
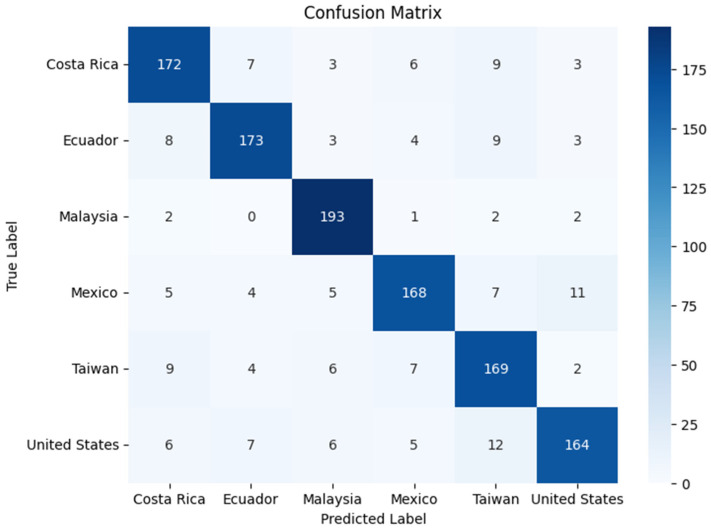
Confusion matrix heatmap representing model performance across five independent Monte Carlo Cross-Validation (MCCV) runs.

As depicted in the confusion matrix of Figure A6 of Appendix A, the model correctly classifies most of the samples across all six countries: Costa Rica, Ecuador, Malaysia, Mexico, Taiwan and the United States. The diagonal elements represent the number of correctly classified data points for each class, while the elements off the diagonal indicate misclassification. The model demonstrates strong classification performance, with the highest accuracy observed for Malaysia (193 correct classifications) followed by Ecuador and Costa Rica (173 and 172 correct classification, respectively).

**Figure A7:**
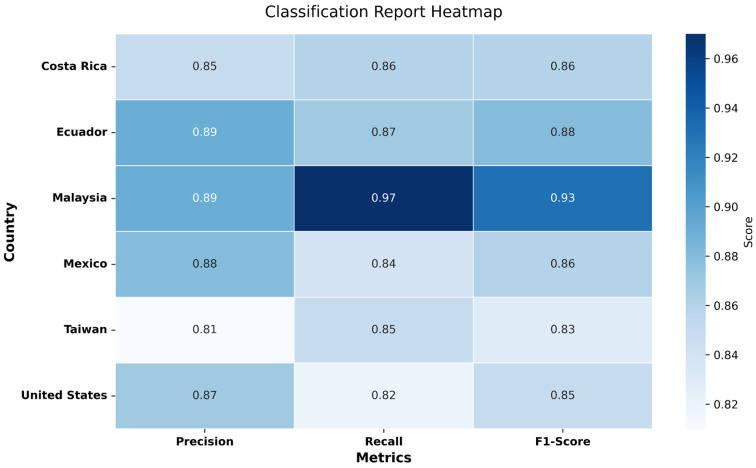
Heatmap of per-class evaluation metrics (Precision, Recall, F1-Score) for the MCCV model.

Figure A7 of Appendix A showcases the evaluation metrics for each predicted class individually. Overall, the F1-Scores ranged from 0.83 to 0.93, showing a strong predictive power of the model. The highest precision value was observed for the class ‘Malaysia’, indicating that the most data points of this class were correctly classified. Also, Malaysia had the highest F1-Score value of 0.93 balancing precision and recall. On the contrary, Taiwan had the lowest precision of 0.81 and a recall of 0.85, suggesting that this class experienced more misclassification than the others.

**Figure A8:**
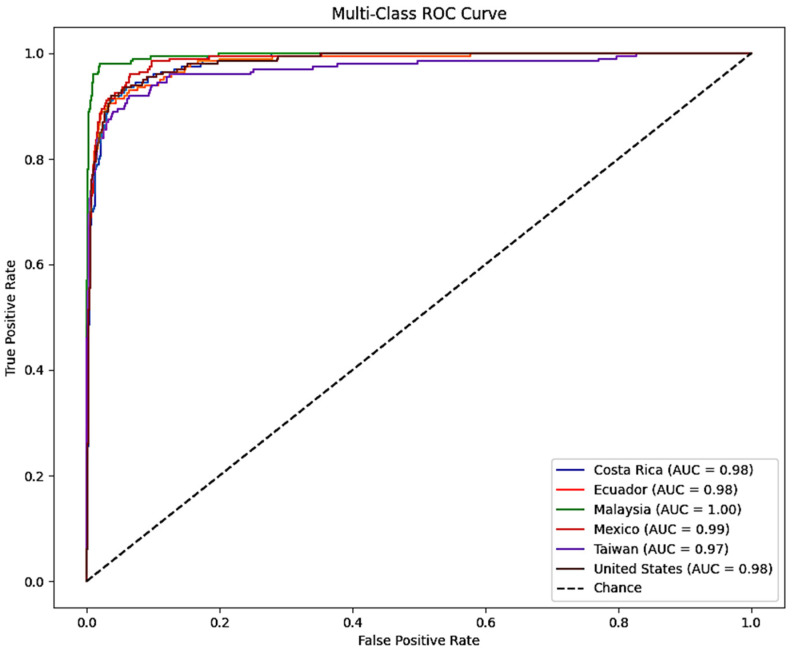
Multi-Class Receiver Operating Characteristic (ROC) curves evaluating the MCCV model’s diagnostic ability.

Figure A8 in the Appendix A presents the multi-class ROC curve, which evaluates the model’s ability to distinguish between different country classes. The Area Under the Curve (AUC) values range from 0.97 to 1.00, indicating a strong predictive capability across all categories. As observed before the class Malaysia is predicted almost flawlessly followed by Ecuador, Costa Rica and Mexico. Overall, the figure suggests that the model has a strong predictive performance.

**Figure 7:**
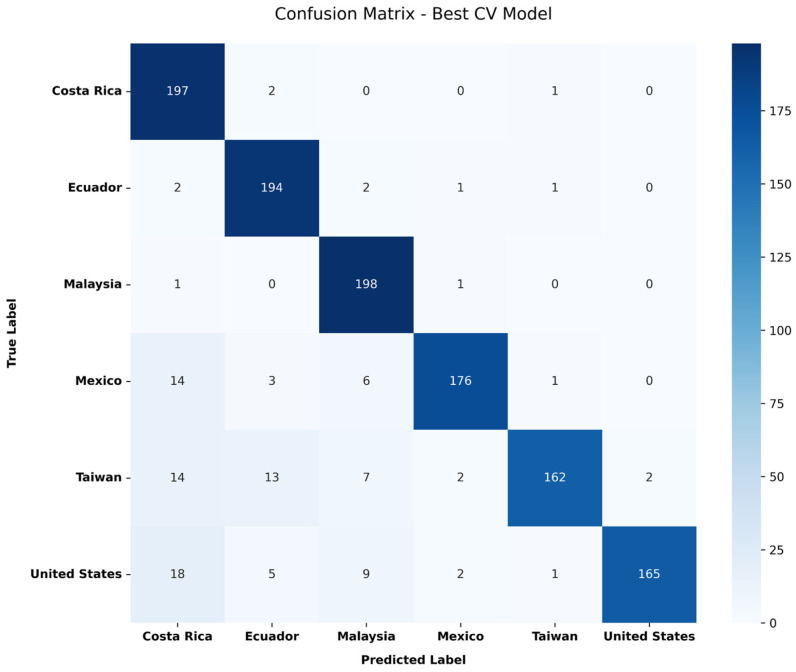
Confusion matrix for the best-performing model derived from 5-fold Cross-Validation.

While the five independent runs provided a strong indication of model performance, a more rigorous evaluation was necessary to ensure true generalization. Therefore, a 5-fold Cross-Validation was conducted to assess model robustness across different data splits. The average accuracy yielded was 0.77 with a standard deviation of ±0.01, and the best-performing model had an accuracy of 0.79. Below, Figure 7 illustrates the performance of the best cross-validated model, Malaysia (198 correct classifications) has the highest predictive power, followed by Costa Rica (197 correct classifications) and Ecuador (194 correct classifications).

**Figure 8:**
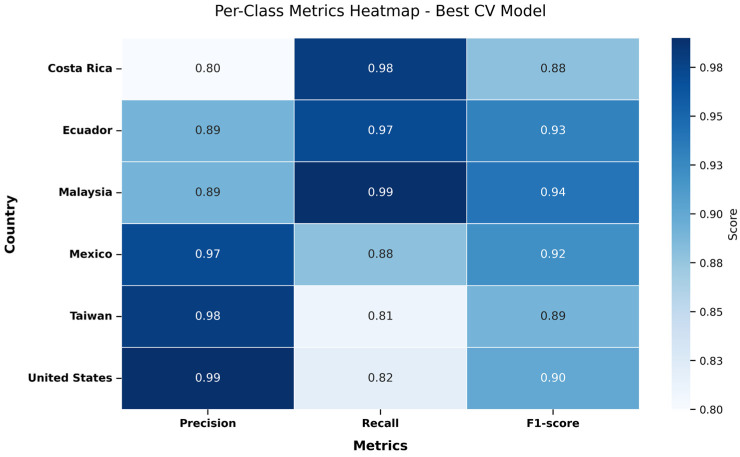
Heatmap of per-class evaluation metrics for the best 5-fold Cross-Validation model.

Figure 8 provides an overview of the model’s performance across classes. Malaysia exhibits the highest recall and F1-Score values, indicating that the model identifies Malaysian sample effectively. In terms of precision, the United States and Mexico demonstrate the highest values, indicating few false-positive predictions. On the other hand, Costa Rica shows the lowest precision, suggesting that samples from other countries are often classified as Costa Rica. Taiwan has the lowest recall, indicating that many samples were misclassified as other countries. Overall, F1-Scores range from 0.88 to 0.94, confirming strong predicting ability across all classes.

**Figure 9:**
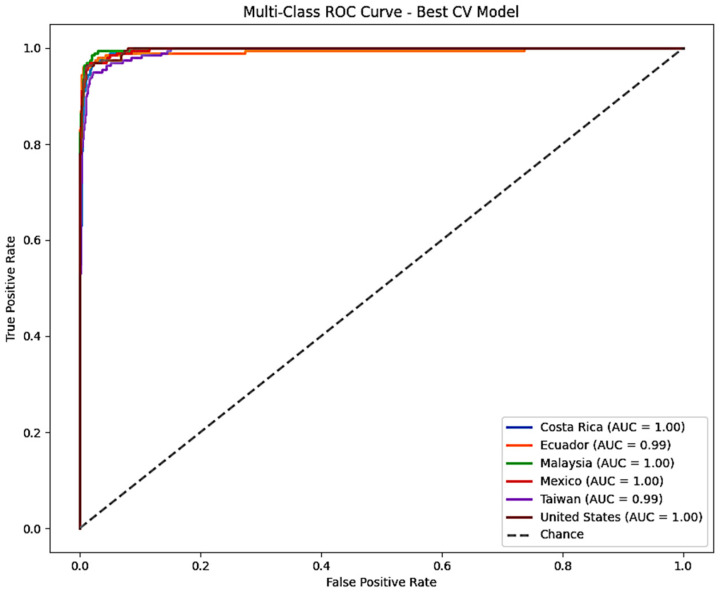
Multi-class ROC curves for the best 5-fold Cross-Validation model.

Figure 9 showcases the model’s ability to correctly distinguish the six countries. The AUC values range from 0.99 to 1.00, demonstrating an exceptional classification performance. The model perfectly predicts Malaysia, Costa Rica, Mexico, and the United States. Ecuador and Taiwan have a slightly lower value. These results align with the confusion matrix and the per-class metrics above.

**Figure 10:**
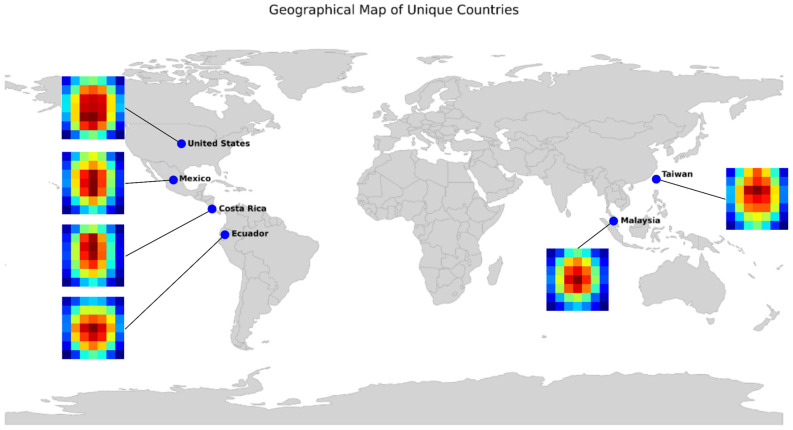
Composite Grad-CAM activation heatmaps for each geographic class. The heatmaps visualize the spatial regions utilized by the model for prediction. Color intensity represents the importance of a specific region of the image to the model’s decision, where red indicates areas of high activation (high influence) and blue indicates areas of low activation (minimal influence).

Beyond numerical evaluation, visual interpretability is crucial to ensuring the model focuses on meaningful image regions. Figure 10 presents the composite Grad-CAM visualizations for the six classification categories. To capture the model’s systematic attention patterns, these heatmaps were generated by aggregating and averaging the activation maps of all correctly classified images within each class. The red-colored areas indicate the regions of highest cumulative activation. Across all six geographic classes, the Grad-CAM analysis reveals a consistent and dominant focus on the central image region. While banana bunches were typically centered during the preprocessing phase, this focal alignment confirms that the classifier has successfully learned to ignore the complex background features (e.g., foliage, soil, and varied lighting) prevalent in the raw field data. By concentrating its activation on the foreground object, the banana bunch, the model demonstrates robust feature extraction that is consistent with the botanical targets of the study. While this interpretation is primarily qualitative and exploratory, the visual evidence across 6000 images suggests that the model’s predictive logic aligns with the foreground object area. However, these activation patterns should be viewed as preliminary observations rather than definitive biological or morphological claims regarding the cultivars.

## 4. Discussion

### 4.1. Reflection on the Results

The results of this study demonstrate that deep learning models can identify regional visual patterns in banana bunches across different countries. However, before evaluating these performance metrics, it is important to define what our “origin” labels truly represent. While the GPS coordinates from iNaturalist provide strong visual context, they do not fully prove that the image was taken at a farm rather than at a local market. For this reason, the geographic labels in this study should be viewed as geolocated botanical observations rather than final proof of a commercial product’s origin. Nevertheless, we believe that checking the fruit at the point of cultivation is the most critical first step for proving its origin. By looking at the fruit where it is actually grown, the model can capture its physical appearance. This approach allows for verification at the very start of the supply chain, before the fruit is packed and shipped; stages where origin labels are most likely to be faked or mixed up.

#### 4.1.1. Findings

Our model performs well in classifying images of bunches of bananas between 6 countries. The starting accuracy was 0.74, rising up to 0.82 after fine-tuning. Finally, with the epoch adjustment, it averaged after 5 runs (MCCV) to 0.86. Although validation accuracy remained relatively stable across Epochs 15, 16, and 17 (hovering around 0.87) as shown in Figure 5, the validation loss demonstrated a clear inflection point (Figure 6). The loss achieved its global minimum at Epoch 15 (0.346) before sharply rising to 0.386 and 0.424 in the subsequent epochs. This divergence between stable accuracy and rising loss indicates that while the model’s classification decisions remained consistent, the calibrated probability estimates were degrading, a sign of early overfitting. Consequently, Epoch 15 was selected as the optimal stopping point to maximize model robustness, resulting in the final configuration detailed in Table 9.

Additionally, this accuracy gap can be attributed to the inherent complexity of the task, as the image dataset exhibits enormous variability as each image is taken from different angles and at different distances with varying image resolution and lighting conditions. To address further the risk of overfitting and obtain a more robust evaluation of our model, a 5-fold Cross-Validation (CV) was conducted. Cross-Validation provided a stricter internal comparison than repeated random splits; however, because the evaluation remained based on the augment-before-split dataset, it does not eliminate the underlying leakage concern and should not be interpreted as a fully realistic estimate of external performance. As expected, the Cross-Validation accuracy was lower compared to the independent runs (0.86), since each fold evaluated the model on unique subsets of data, reducing the impact of lucky train–test splits. Also, the k-fold Cross-Validation ensures that the model is trained and tested on distinct subsets of the dataset in each iteration. In contrast, MMCV does not guarantee that all data points are utilized equally across all runs. Due to the randomness in its design, some samples may be selected for training or testing multiple times while others may not be included at all. Given that the 5-fold CV provides a more generalizable performance estimate, the best-performing model from Cross-Validation (0.79 accuracy) was selected as the final model for interpretation.

Furthermore, our functional ablation study on network capacity highlighted in a complementary way the complexity of the classification task. The underfitting observed with lower-capacity classification heads suggests that the visual differences captured in this dataset are not trivial to separate. Conversely, the rapid overfitting observed in higher-capacity configurations indicates sensitivity to the limited and augmented nature of the dataset. These observations support the existence of learnable image patterns within the experimental corpus, but they do not establish robust generalization to truly unseen original images.

Regarding interpretability, the Grad-CAM analysis (Figure 10) provides a qualitative understanding of the model’s logic by highlighting influential regions in the decision-making process. At the current stage of this proof-of-concept, the analysis remains primarily qualitative, allowing us to observe that the model captures broad morphological traits such as bunch elongation, compaction, and dispersion patterns. For instance, Latin American origins (Ecuador, Costa Rica, and Mexico) exhibit vertical, elongated activation shapes, while Malaysia shows a more compact, circular pattern. These exploratory findings suggest a focus on biologically relevant structural traits rather than random background noise, which was effectively eliminated during the preprocessing and segmentation stages. However, we acknowledge that the current analysis remains primarily qualitative, and definitive proof of morphological capture remains a goal for future research. Transitioning from these qualitative observations to quantitative metrics, such as calculating the Intersection over Union (IoU) between high-activation heatmaps and expert-annotated bounding boxes, would be required to statistically validate these claims.

When compared with laboratory-based approaches such as spectroscopy, chemical profiling, or multimodal sensor fusion, the present RGB-image approach offers potential advantages in accessibility, cost, and scalability because it does not require specialized instrumentation. For instance, while traditional analytical techniques such as Nuclear Magnetic Resonance (NMR) and Mass Spectrometry (MS) provide definitive chemical traceability, they are significantly constrained by high capital investments for instrumentation (often exceeding hundreds of thousands of euros), expensive maintenance, and the need for highly skilled personnel to interpret complex spectral data [25]. Furthermore, these laboratory-based methods typically necessitate destructive, time-consuming sample preparation protocols, such as physical homogenization, extensive solvent extraction, or chromatographic separation, which can require anywhere from 30 min to several hours per batch, thereby hindering rapid, on-site analysis [26]. In contrast, our proposed pipeline relies on non-destructive RGB imagery captured via ubiquitous consumer hardware (smartphones). This approach reduces the marginal cost of data acquisition and sample preparation time to near zero, enabling scalable, high-throughput screening directly at the cultivation stage. However, laboratory-based methods benefit from controlled acquisition conditions and stronger analytical traceability, whereas the current citizen-image framework is highly variable and not yet validated for independent generalization. The two approaches should therefore be viewed as complementary rather than directly interchangeable.

Lastly, the model’s generalizability is currently restricted to a single cultivar. It is highly likely that morphological markers will vary across different cultivars, requiring multi-cultivar datasets in the future. Furthermore, while Grad-CAM visualization provides qualitative insight, definitive proof that the model captures meaningful morphological differences requires quantitative metrics, such as the Intersection over Union between high-activation heatmaps and expert-annotated bounding boxes.

#### 4.1.2. Limitations

There are several noteworthy limitations to this study. A major conceptual limitation concerns the interpretation of the geographic label. In the present study, the label derives from the location attached to an iNaturalist observation rather than from independently verified supply-chain documentation. As a result, the task is more accurately described as classification of geolocated banana observations than as definitive food origin authentication. This distinction is important when interpreting the relevance of the results to food fraud applications.

In addition, the images require substantial preprocessing, which demands significant computational resources. If such resources are not readily available, this requirement may hinder both the performance and scalability of the project.

Moreover, the quality of the images plays a critical role. In this study, many images were discarded due to poor resolution or other quality-related issues. If those images were meeting acceptable quality standards and they were retained, the model might have adapted better to real-world conditions.

While standard practice in image classification is to partition a dataset prior to augmentation, this study utilized an ‘augment-before-split’ protocol to address the constraints of a limited dataset. Given an initial foundation of approximately 150 original observations per geographic class, a pre-augmentation split would have yielded a test set too small for statistically significant analysis (*n* ≈ 30). To ensure a dense feature space for manifold learning and a robust evaluation consisting of 200 test images per class, the 80/20 split was performed on the fully augmented set [27]. While this approach can introduce a risk of data leakage, to minimize the associated risk, high-variance geometric transformations were applied to ensure that all augmented instances represented distinct visual data points [20].

Therefore, the reported accuracy should be understood as the model’s ability to generalize within this high-variance domain. This represents a deliberate choice to prioritize a large, stable test sample over absolute data independence, ensuring our performance metrics are statistically significant. Because augmented samples derived from the same original image likely appeared in both the training and testing sets, our metrics overestimate true real-world generalization. Future studies could refine this evaluation by employing group k-fold splitting, which confines all augmented variants of a single original image to the same fold, thereby strictly enforcing independence [18], since standard k-fold cross validation misrepresent variance when data dependencies exist [28,29]. Furthermore, a major conceptual limitation is our assumption that iNaturalist GPS coordinates correspond to the true origin of a food product. These coordinates represent botanical cultivation sites. While it is valid for origin tracking at the production level, this does not automatically equate to the origin of processed and packages commercial bananas in supply chains. Additionally, incorporating photometric augmentations, such as color jittering, brightness shifts, or noise injection, would further challenge the model’s robustness against variable lighting conditions, likely yielding more conservative but environmentally generalizable performance metrics [20].

Additionally, another limitation concerns the selection of the base model. In our study, we focus on lightweight models due to computational constraints. MobileNetV1 is an older architecture, lightweight model optimized for speed and reduced memory usage. While this choice was necessary given the available resources, more complex models could have yielded higher performance and generalization. Also, the input of the image of the model is of low definition as the input that the model can receive is 224 × 224 pixels. To meet this requirement, all images were downsized while trying to retain as much information as possible. This could have a limitation on the performance of our model.

A further challenge lies in the heterogeneity of the images even in the same cultivar represented in the dataset. In our case, we concentrated only on one cultivar, but since the images in iNaturalist are uploaded by the observers, there is a potential risk of misclassification. These possibly misclassified images may introduce noise to the dataset and influence the model’s accuracy. Additionally, over half of our dataset was artificially generated through the data augmentation process rather than consisting of original images. If a larger number of unique images were available, the level of heterogeneity would be even greater.

Furthermore, the dataset used in our study depicts bunches of bananas rather than individual fruits. This approach aligns with a production’s/farmer’s level rather than a consumer-level analysis. If future research is targeted at the consumer level, new data-collection strategies must be applied. Citizens should upload multiple high-definition images of the same fruit from different angles, adding a practical limitation on the qualitative consistency of the uploaded images.

Lastly, the generalizability of the present findings is further limited by the narrow scope of the dataset, which includes only one cultivar (Cavendish), one image source platform, and six countries selected after data reduction. Accordingly, the current results do not support claims of transferability across cultivars, broader geographic diversity, processed banana products, retail settings, or supply-chain authentication scenarios. Nevertheless, the proposed pipeline was designed in a modular manner and is methodologically transferable. The data-collection and preprocessing stages rely on adaptable textual prompts, meaning that the same workflow could be reconfigured for other cultivars or fruit species by altering the prompt definitions and reconstructing the image set. Similarly, the downstream modeling stage, screening, transfer learning, and model selection, follow a generic framework that can be reused in related visual classification tasks. Such transferability should be understood as procedural rather than empirical, as it was not validated beyond the present case study.

Overall, while our approach shows the potential of applying DL in bananas’ country of origin classification, the limitations on data collection, data quality, model selection and computational resources are necessary for future studies.

## 5. Conclusions

This research illustrates the potential of deep learning (DL) coupled with citizen science as an exploratory approach for investigating origin-related visual discrimination in bananas from images.

Although this study focuses on banana bunches in natural environments rather than commercial packaged products, it provides a proof-of-concept at the cultivation–observation level. The study shows that CNN-based models can learn origin-related visual patterns from a refined citizen-generated image dataset. However, because augmentation was applied prior to dataset splitting, the reported performance metrics should be interpreted as upper-bound estimates rather than as evidence of true independent generalization. In addition, the findings are limited by the use of a single cultivar, one image source platform, and six countries, and therefore do not support direct claims of transferability to other cultivars, products, or real-world supply-chain authentication scenarios.

Future research could investigate consumer-level use cases in which individual bananas, rather than bunches, are used as model inputs. Such an extension would require dedicated dataset construction and model development for individual-fruit classification. If lightweight architectures remain effective under leakage-free validation, their eventual integration into mobile or edge-based systems may be explored for rapid image-based screening in field settings.

Future studies may also compare lightweight models with more complex deep learning architectures to determine whether increased computational complexity yields improved robustness and classification performance.

They should also prioritize larger datasets with more original images per country, reduced reliance on augmentation, and broader geographic coverage. Additional banana cultivars could also be incorporated to test whether the workflow can be extended across varietal diversity. More broadly, the methodological pipeline may be adaptable to other fruit species, such as avocado or mango, provided that sufficiently large and well-curated citizen-generated image datasets are available.

Because citizen science plays a central role in the proposed framework, future research should also investigate structured data-collection campaigns in which contributors follow standardized image-acquisition protocols. Guidance on lighting conditions, image angles, framing, and metadata inclusion could improve data quality, reduce noise, and support more robust model development.

## Figures and Tables

**Table 1:** Example of location data including inconsistent formatting before and after processing.

Original Location Data	Processed Location Data
Kuching, MY-SK, MY	Malaysia
New York Ave, Corpus Christi, TX, US	United States
Puerto Rico	Puerto Rico
SÃ£o Paulo—SP, 08253, Brasil	Brazil
Avenida 0, Turrialba, Cartago, CR	Costa Rica
Mesa Verde Dr, Saint Cloud, FL, US	United States
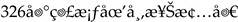	Taiwan
SÃ£o Miguel Island, Nordeste, Azores, PT	Portugal
Arroio do Sal, RS, 95585-000, Brasil	Brazil
UnB Posto EcolÃ^3^gico—BrasÃlia, DF, Brasil	Brazil
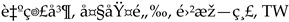	Taiwan

**Table 2:** Image count per country.

	Country	Image Count
**1**	Taiwan	199
**2**	United States	195
**3**	Mexico	172
**4**	Ecuador	161
**5**	Malaysia	154
**6**	Costa Rica	152
**7**	Colombia	131
**8**	Puerto Rico	116
**9**	Brazil	100
**10**	Panama	90
**11**	Bolivia	63
**12**	Spain	60
**13**	Thailand	55
**14**	Indonesia	53
**15**	Portugal	40
**16**	Peru	39
**17**	Singapore	35
**18**	India	31
**19**	Dominican Republic	25
**20**	China	24

**Table 3:** Data augmentation parameters.

Parameter	Value	Description
Rotation Range	±20 degrees	Introduces variations in image orientation
Horizontal and Vertical shifts	±20%	Simulates changes in the position of objects
Shear Transformation	20%	Applies perspective-like distortions
Zoom Range	±20%	Adjusts the scale for zooming effects
Horizontal Flipping	True	Generates mirrored versions of original images
Fill mode	True	Smoothly fills empty pixels

**Table 4:** Benchmarking of MobileNet models.

Model	Test Accuracy	Test Loss
MobileNetV1	0.74	0.75
MobileNetV2	0.67	0.90
MobileNetV3Small	0.19	1.79
MobileNetV3Large	0.21	1.76

**Table 5:** Baseline model configuration.

Input Size	Optimizer	Learning Rate	Loss Function	Evaluation Metric	Batch Size	Epochs	Early Stopping	Learning Rate Scheduler
224 × 224	RMSprop	0.001	sparse categorical cross-entropy	accuracy	64	10	3	0.2

**Table 6:** Ablation study of classification head capacity (Fixed Batch Size: 32, Dropout: 0.2).

Dense Units	Validation Accuracy	Validation Loss
64	0.4925	1.311
128	0.7750	0.625
256	0.8217	0.498

**Table 7:** Best model configuration.

Val Accuracy	Val Loss	Batch Size	Dense Units	Dropout Rate	Epochs	Learning Rate
0.8217	0.498	32	256	0.2	10	0.001

**Table 8:** Accuracy over different numbers of epochs.

Epochs	Test Accuracy	Test Loss
10	0.8217	0.498
18	0.8667	0.095

**Table 9:** Final model summary.

Average Test Accuracy	Batch Size	Dense Units	Dropout Rate	Epochs	Learning Rate
0.878	32	256	0.2	15	0.001

## Data Availability

The original contributions presented in the study are included in the article/supplementary material, further inquiries can be directed to the corresponding author.

## Supplementary Materials

The following supporting information can be downloaded at: https://www.mdpi.com/article/10.3390/foods15101628/s1. Table S1: MobileNetV1’s architecture; Table S2: Complete Hyperparameter Grid Search Results.

## Abbreviations

The following abbreviations are used in this manuscript:

AI	Artificial Intelligence
API	Application Programming Interface
AUC	Area Under the Curve
CNN	Convolutional Neural Network
CPU	Central Processing Unit
CSV	Comma-Separated Values
CV	Cross-Validation
DA	Discriminant Analysis
DL	Deep Learning
EU	European Union
GAP	Global Average Pooling
GLM	Generalized Linear Model
GPS	Global Positioning System
GPU	Graphics Processing Unit
Grad-CAM	Gradient-weighted Class Activation Mapping
MCCV	Monte Carlo Cross-Validation
NIR	Near-Infrared
NMR	Nuclear Magnetic Resonance
NN	Neural Network
RF	Random Forest
RGB	Red, Green, Blue
ROC	Receiver Operating Characteristic
SAM	Segment Anything Model
SVM	Support Vector Machine
URL	Uniform Resource Locator

## Appendix A

**Table A1:** Confusion Matrix of Grounded-DINO model.

	Predicted: Banana	Predicted: No Banana
**Actual: Banana**	3935 (TP)	63 (FN)
**Actual: No Banana**	2266 (FP)	1865 (TN)

**Table A2:** Different Types of subsets.

Subset 1			Subset 2			Subset 3		
Dataset_50			Dataset_100			Dataset_150		
	Country	Image_Count		Country	Image_Count		Country	Image_Count
**1**	Taiwan	199	**1**	Taiwan	199	**1**	Taiwan	199
**2**	United States	195	**2**	United States	195	**2**	United States	195
**3**	Mexico	172	**3**	Mexico	172	**3**	Mexico	172
**4**	Ecuador	161	**4**	Ecuador	161	**4**	Ecuador	161
**5**	Malaysia	154	**5**	Malaysia	154	**5**	Malaysia	154
**6**	Costa Rica	152	**6**	Costa Rica	152	**6**	Costa Rica	152
**7**	Colombia	131	**7**	Colombia	131	**Total**		1033
**9**	Brazil	100	**9**	Brazil	100			
**10**	Panama	90	**Total**		1264			
**11**	Bolivia	63						
**12**	Spain	60						
**13**	Thailand	55						
**14**	Indonesia	53						
**Total**		1585						

**Table A3:** Model comparison [30].

Model	Size (MB)	Parameters (M)	Depth (Layers)	Top 5 Accuracy
MobileNetV2	14	3.5	105	0.901
MobileNet	16	4.3	55	0.895
NASNetMobile	23	5.3	389	0.919
EfficientNetB0	29	5.3	132	0.933
EfficientNetV2B0	29	7.2	-	0.943
EfficientNetB1	31	7.9	186	0.944
DenseNet121	33	8.1	242	0.923
EfficientNetV2B1	34	8.2	-	0.950
EfficientNetB2	36	9.2	186	0.957

**Table A4:** Benchmarking of pre-trained models across all datasets.

Subset	50 × 400	50 × 1000	100 × 400	100 × 1000	150 × 400	150 × 1000
Model	Accuracy					
MobileNetV2	0.46	0.62	0.45	0.63	0.51	0.68
MobileNetV1	0.48	0.68	0.46	0.64	0.51	0.73
NASNetMobile	0.32	0.45	0.32	0.41	0.45	0.54
EfficientNetB0	0.07	0.07	0.11	0.11	0.17	0.17
EfficientNetV2B0	0.07	0.07	0.11	0.11	0.17	0.17
EfficientNetB1	0.07	0.07	0.11	0.11	0.17	0.17
DenseNet121	0.37	0.56	0.38	0.51	0.48	0.60
EfficientNetV2B1	0.07	0.07	0.11	0.11	0.17	0.17
EfficientNetB2	0.07	0.07	0.11	0.11	0.17	0.17
