# Supplementary material for: Food Origin Authenticity Using Deep Learning and Citizen Science: Bananas Case Study
Source: Foods. 2026 May 7;15(10):1628. doi: 10.3390/foods15101628 (PMC13205795; doi:10.3390/foods15101628)
Supplement: Supplementary file 1 [file foods-15-01628-s001.zip › foods-4207842-supplementary.pdf]

Table S 1. MobileNetV1's architecture

Type / Stride	Filter Shape	Input Size
Conv / s2	3 × 3 × 3 × 32	224 × 224 × 3
Conv dw / s1	3 × 3 × 32 dw	112 × 112 × 32
Conv / s1	1 × 1 × 32 × 64	112 × 112 × 32
Conv dw / s2	3 × 3 × 64 dw	112 × 112 × 64
Conv / s1	1 × 1 × 64 × 128	56 × 56 × 64
Conv dw / s1	3 × 3 × 128 dw	56 × 56 × 128
Conv / s1	1 × 1 × 128 × 128	56 × 56 × 128
Conv dw / s2	3 × 3 × 128 dw	56 × 56 × 128
Conv / s1	1 × 1 × 128 × 256	28 × 28 × 128
Conv dw / s1	3 × 3 × 256 dw	28 × 28 × 256
Conv / s1	1 × 1 × 256 × 256	28 × 28 × 256
Conv dw / s2	3 × 3 × 256 dw	28 × 28 × 256
Conv / s1	1 × 1 × 256 × 512	14 × 14 × 256
5× Conv dw / s1	3 × 3 × 512 dw	14 × 14 × 512
Conv / s1	1 × 1 × 512 × 512	14 × 14 × 512
Conv dw / s2	3 × 3 × 512 dw	14 × 14 × 512
Conv / s1	1 × 1 × 512 × 1024	7 × 7 × 512
Conv dw / s2	3 × 3 × 1024 dw	7 × 7 × 1024
Conv / s1	1 × 1 × 1024 × 1024	7 × 7 × 1024
Avg Pool / s1	Pool 7 × 7	7 × 7 × 1024
FC / s1	1024 × 1000	1 × 1 × 1024
Softmax / s1	Classifier	1 × 1 × 1000

Table S 2. Complete Hyperparameter Grid Search Results

Val Accuracy	Val Loss	Batch Size	Dense Units	Dropout Rate	Epochs	Learning Rate
82.17%	0.498	32	256	0.2	10	0.001
81.83%	0.530	16	256	0.2	10	0.0005
80.75%	0.562	16	256	0.3	10	0.0005
80.67%	0.570	32	256	0.2	10	0.0005
80.25%	0.574	64	256	0.2	10	0.001
79.92%	0.609	32	256	0.3	10	0.0005
79.42%	0.587	32	256	0.3	10	0.001
79.00%	0.664	32	128	0.2	10	0.0005
78.58%	0.623	64	256	0.3	10	0.001
78.42%	0.584	16	256	0.3	10	0.001
77.83%	0.638	16	128	0.2	10	0.0005
77.83%	0.608	16	256	0.2	10	0.001
77.58%	0.680	64	128	0.2	10	0.001
77.50%	0.625	32	128	0.2	10	0.001
76.83%	0.696	64	256	0.3	10	0.0005
76.67%	0.665	64	256	0.2	10	0.0005
75.50%	0.737	32	128	0.3	10	0.0005
75.42%	0.717	16	128	0.3	10	0.0005

75.33%	0.741	32	256	0.5	10	0.0005
74.92%	0.708	32	128	0.3	10	0.001
74.50%	0.741	16	256	0.5	10	0.0005
74.08%	0.704	16	128	0.2	10	0.001
72.58%	0.764	16	128	0.3	10	0.001
71.67%	0.815	64	256	0.5	10	0.0005
71.08%	0.855	64	128	0.3	10	0.0005
70.67%	0.814	32	256	0.5	10	0.001
70.67%	0.822	64	128	0.2	10	0.0005
70.50%	0.797	64	128	0.3	10	0.001
70.50%	0.807	16	64	0.2	10	0.0005
70.33%	0.807	64	256	0.5	10	0.001
69.92%	0.805	16	256	0.5	10	0.001
69.42%	0.878	16	64	0.3	10	0.0005
69.33%	0.842	32	64	0.2	10	0.0005
69.33%	0.921	16	256	0.2	10	0.0001
69.25%	0.828	32	64	0.2	10	0.001
68.92%	0.831	16	64	0.2	10	0.001
68.00%	0.961	16	256	0.3	10	0.0001
67.92%	0.928	32	128	0.5	10	0.0005
67.58%	0.876	32	64	0.3	10	0.001
67.25%	0.888	64	64	0.2	10	0.001
67.08%	0.884	16	128	0.5	10	0.0005
66.75%	0.918	32	64	0.3	10	0.0005
65.83%	0.942	16	128	0.5	10	0.001
65.75%	0.959	64	64	0.3	10	0.001
65.42%	0.971	64	128	0.5	10	0.001
65.33%	0.923	32	128	0.5	10	0.001
65.25%	0.982	64	64	0.2	10	0.0005
65.00%	0.983	64	64	0.3	10	0.0005
64.75%	1.017	16	64	0.5	10	0.0005
64.17%	1.007	64	128	0.5	10	0.0005
62.67%	1.051	32	256	0.2	10	0.0001
62.42%	1.064	16	256	0.5	10	0.0001
62.08%	0.989	16	64	0.3	10	0.001
61.58%	1.118	16	128	0.3	10	0.0001
61.25%	1.095	32	256	0.3	10	0.0001
60.17%	1.142	64	64	0.5	10	0.0005
59.33%	1.101	32	64	0.5	10	0.0005
58.83%	1.089	16	128	0.2	10	0.0001
57.67%	1.185	32	128	0.2	10	0.0001

57.42%	1.221	16	128	0.5	10	0.0001
57.25%	1.200	64	256	0.3	10	0.0001
57.17%	1.201	32	128	0.3	10	0.0001
56.92%	1.193	32	256	0.5	10	0.0001
56.33%	1.182	64	64	0.5	10	0.001
56.08%	1.192	64	256	0.2	10	0.0001
56.08%	1.177	32	64	0.5	10	0.001
55.42%	1.219	16	64	0.3	10	0.0001
54.25%	1.224	16	64	0.2	10	0.0001
53.08%	1.269	32	128	0.5	10	0.0001
51.92%	1.246	16	64	0.5	10	0.001
51.42%	1.318	16	64	0.5	10	0.0001
51.33%	1.292	64	256	0.5	10	0.0001
50.75%	1.332	32	64	0.3	10	0.0001
50.25%	1.307	64	128	0.2	10	0.0001
49.50%	1.322	64	128	0.3	10	0.0001
49.25%	1.312	32	64	0.2	10	0.0001
47.50%	1.404	32	64	0.5	10	0.0001
47.08%	1.377	64	64	0.2	10	0.0001
46.92%	1.405	64	128	0.5	10	0.0001
44.67%	1.424	64	64	0.3	10	0.0001
41.67%	1.515	64	64	0.5	10	0.0001
